# Working Memory and Divergent Thinking: The Moderating Role of Field-Dependent-Independent Cognitive Style in Adolescence

**DOI:** 10.3390/bs13050397

**Published:** 2023-05-10

**Authors:** Marco Giancola, Simonetta D’Amico, Massimiliano Palmiero

**Affiliations:** 1 Department of Biotechnological and Applied Clinical Sciences, University of L’Aquila, 67100 L’Aquila, Italy; 2 Department of Communication Sciences, University of Teramo, 64100 Teramo, Italy

**Keywords:** executive functions, creativity, cognitive style, moderation, adolescents

## Abstract

Divergent thinking (DT) is considered a key process of creativity. It is supported by different mental processes, ranging from executive functions to cognitive styles. The extent to which these processes jointly contribute to DT is still unclear, especially in adolescence, which represents a developmental stage that involves fundamental changes and restructuring in cognition, emotion, and personality. The present study hypothesises that the field-dependent-independent cognitive style (FDI) moderates the relationship between working memory capacity (WMC). A convenient sample of one hundred adolescents (mean age 18.88 years) was tested in terms of FDI by the Embedded Figure Test (EFT), which requires finding a simple shape as fast as possible within a complex figure. WMC was assessed by the Digit Span Forward Test (DSFT), which requires recalling sequences of numbers in the same order immediately after the presentation. DT was assessed by the Alternative Uses Test (AUT), which requires finding as many uses as possible for common objects. The main result was that the field-independent cognitive style (FI) positively moderated the effect of WMC on DT. This result extends previous findings on the critical role of FDI in real-world creativity, suggesting that FI adolescents better exploit the effect of WMC on DT by using more analytic and associative strategies, focusing on relevant elements when facing a problem, and retrieving conceptual knowledge more efficiently. Implications, limits, and future research directions are briefly discussed.

## 1. Introduction

Divergent thinking (DT) has gained increasing attention in recent years, as it has been proposed as a tool for promoting well-being in different contexts, such as educational, clinical, and ecological contexts, e.g., Refs. [1,2]. The success of DT is based on the idea that it is viewed not only as an index of creative potential [3] but also as a strategy to solve ill-defined problems [4]. Indeed, DT reflects the ability to find alternative solutions to open-ended problems, breaking the schemes and trying to be as creative as possible [5,6]. DT has been defined as a goal-directed process [7] supported by default, salience, and executive systems [8,9]. As concerns the latter, working memory seems to play a critical role in DT, as it increases the ability to convey multiple pieces of information into the stream of attention, enhancing the likelihood of finding remote connections [10]. Furthermore, given its multifaceted nature, DT is supported by many other factors, including cognitive styles, which reflect information processing strategies such as the field-dependent-independent cognitive style (FDI). The latter lies in the view that individuals have different degrees of dependence on the surrounding environment [11]. This means that field-independent people (FIs) are less constrained from the field, being able to perceive simple figures embedded into more complex figures more easily. By contrast, field-dependent people (FDs) are more constrained from the field and, consequently, perceive simple figures as complex ones less easily. Previous studies have shown a significant association between FDI and DT [12], suggesting the pivotal role of field-independence (FI). However, previous research [13] proposed that working memory, as a high-order cognitive process, is modulated by FDI. This opens the idea that DT might represent the result of the joint effect of cognitive processes (i.e., working memory capacity (WMC)) and information processing strategies (i.e., field-independent cognitive style). In this vein, the investment theory of creativity [14] assumes the interaction of different individual resources, including cognitive processes and cognitive styles [15], as primary determinants of creativity. Notably, the current research focuses on adolescence, which represents a developmental stage involving profound changes and restructuration in cognition, including DT [16] and working memory [17], through increasing knowledge and the coordination of associative and analytic processes [18]. Regarding information processing strategies underpinned by FDI, research showed that this cognitive style develops since childhood, even though the trend is not necessarily linear, reaching a plateau around the age of 17, that is, during adolescence [19]. Accordingly, in a sample of adolescents, the current research addressed the moderating role of FDI in the association between WMC and DT.

### 1.1. Working Memory Capacity and Divergent Thinking

WMC is involved in goal-directed behaviours in which information in a different format can be held, manipulated, and updated [20]. It allows for a small amount of information to be used for other cognitive tasks, such as thought, reasoning, problem-solving, decision-making, and language. WMC also relies on other functions, including focused attention [21] and the ability to combine multiple types of information [22]. Noteworthily, some previous studies showed that WMC (e.g., span) does not affect DT [23,24,25] or marginally affects DT [26]. For example, children with a high WMC showed poorer DT performance than children with low WMC [26]. In soccer athletes, the domain-general WMC, as measured by solving math problems while trying to remember an unrelated set of letters, was found to be unrelated to a soccer-specific DT test, which consisted of finding many different alternative solutions to offensive football scenes [27]. Even though these results question the association between WMC and DT, other studies revealed that WMC is directly related to different components of DT, such as fluency [28,29], flexibility [30], elaboration [31], originality, infrequency, and persistence [28], or indirectly related to DT by intelligence [32], associative fluency [33], and task instructions [34]. Specifically, increasing WMC can support the extraction of information while performing DT activities [35]. Similarly, people with high WMC performed better in visual and verbal DT tasks, except for visual fluency [36]. Additionally, working memory updating, which is strongly predicted by WMC [37,38], was also found to play a critical role in DT [39], mainly in terms of fluency [40,41]. The view that high WMC contributes to DT by enhanced persistence and consequently focused attention is noteworthy [28], whereas low working memory and consequent defocused attention by enhanced flexibility [42] is likely. In this vein, in a large sample (1221 subjects), the originality and fluency of DT were associated with greater brain activity during working memory updating in the ventral attention system in the right hemisphere and reduced task-induced deactivations of the default mode network, suggesting alterations in attentional reallocation [43]. However, working memory updating training yielded reductions in activation in the ventrolateral and dorsolateral prefrontal cortex, two brain areas that play a critical role in DT [44]. Furthermore, evidence showed an enhancement of fluency, originality, and elaboration after 13 weeks of WMC training [45]. Altogether, these results show that, even though some studies found that working memory and DT are unrelated, the two abilities share some common variance based on other processes. 

### 1.2. Field-Dependent-Independent Cognitive Style and Divergent Thinking

The association between FDI and creativity has been a focus of research in psychology, given the pivotal role of cognitive styles in acquiring, organizing, and using information for different goals in everyday situations [46]. Although FIs have mainly been defined as more flexible, open-minded, and more capable of breaking down the routine than FDs, empirical evidence on the role of FDI on creativity provided puzzled results in different domains of creativity, including creative potential measures (i.e., divergent thinking, convergent thinking, and creative personality) and real-world creative production [47]. Indeed, performance in the Embedded Figure Test (EFT) was negatively correlated with different measures of visual DT in terms of fluency, flexibility, and originality; moreover, no significant associations between FDI (as captured by the Hidden Figure Test) and verbal DT were found [48]. Similarly, no significant differences between FIs and FDs in the Torrance Test of Creative Thinking were found in a sample of 141 high school students [49]. Despite this evidence, a set of recent studies suggested that FI is deeply involved in different domains of creative performance. For instance, FIs were found to outperform FDs in the ability to generate an original and appropriate object in a real-world context [50]. Additionally, FIs were found to show higher scores than FDs in scientific and social idea generation in terms of fluency and novelty [51], while a significant main effect of FDI was observed for both fluency and the originality scores of verbal DT [52]. Similarly, high performance in the Group Embedded Figure Test was associated with high levels of elaboration in the Torrance Test of Figural Creativity [53]. Finally, FDI was revealed to be a critical factor in both creative potential and real-world creative production [12], suggesting that FIs’ ability to apply more analytical strategies to solve problems and break down an issue into small parts represents a key driver for the generation of appropriate and original ideas. Altogether, these findings leave room for a reasonable involvement of field-independent cognitive style in creative performance.

### 1.3. The Moderating Role of Field-Dependent-Independent Cognitive Style

Overall, FDs are sensitive to the surrounding environment and are externally goal-oriented [54,55]. In other words, they rely on the context in which they are embedded. FIs are not sensitive to the surrounding environment and are insensitive to social concerns and internal goal orientation [56]. These findings suggest that FDI significantly affects people’s everyday life, affecting their performance in different domains, including social, emotional, and cognitive domains. As concerns the latter, prior studies highlighted that FDI modulates individual performance in tasks that require high-order cognitive processes, including DT [47,50] and WMC [57], even under high load conditions [13]. This is in line with the finding that FI children 8–11 years old outperformed FD children in terms of verbal working memory [58]. However, FD students with low verbal working-memory capacity performed worse in mathematics, whereas FI students that used their working-memory capacity more efficiently performed better [59]. This scenario offers theoretical grounds to assume that FDI and working memory jointly affect DT. Therefore, drawing upon the investment theory of creativity [14], the current research hypothesis was formulated as follows—FDI moderates the association between WMC and DT.

## 2. Materials and Methods

### 2.1. Participants and Procedure

One hundred healthy Italian adolescents (mean_age_ = 18.88 years; SD_age_ = 0.32 years; 50 females) participated in this research. All participants were selected randomly from the University of L’Aquila, Italy. They were students in the first year of the bachelor’s degree in psychology, motor and sports sciences, and biotechnology. All subjects participated voluntarily, and no rewards were offered. Participants were recruited as follows: an invitation was disseminated online through university e-mail, and students were requested to confirm their availability if they were between 18–19 years old. Through this procedure, participants were recruited randomly upon availability. All participants signed the informed consent and completed a socio-demographic questionnaire assessing age, gender, general health, and educational level. Following that, subjects were requested to perform the Digit Span Forward Test, the Embedded Figure Test, and the Alternative Uses Task. The order of the measures was randomized. Based on the socio-demographic questionnaire, no subjects reported psychiatric and neurological disorders or drug and alcohol addiction. Furthermore, no participants reported having a background or formal achievement in art. The experiment was conducted in a quiet room of the Socio-Cognitive Processes in Life Span Laboratory at The University of L’Aquila (L’Aquila, Italy). The whole experiment lasted approximately 45 min. The Local Ethics Committee approved this research in accordance with the Declaration of Helsinki.

### 2.2. Measures

The Digit Span Forward Test (DSFT) [60] represents the golden measure of WMC. It requires participants to recall, in the same order, a series of digits immediately after the presentation. The experimenter said the numbers aloud at a rate of one per second. The sequence of numbers varied from two to nine digits. After two consecutive errors, the task was interrupted, and the participant’s span was the last sequence of numbers correctly repeated at least once.

The Embedded Figure Test (EFT) [61] is a paper and pencil test in which participants were requested to find a simple black and white shape within a geometric colored complex figure. Specifically, the EFT consists of 24 cards (12 cards with simple shapes and 12 cards with complex figures) that were 12.9 × 7.7cm. The experimenter presented the complex-colored figures one by one for 15 s, and the participant had to describe the figure loudly. Following that, the experimenter removed the complex figure and presented the simple one. After 10 s, the simple black and white shape was hidden, and the experimenter presented the complex-colored figure again. Afterwards, participants were asked to find the simple black and white shape embedded in the complex figure, tracing the outlines using a pencil. When the participants declared that they had found the simple black and white shape within the complex figure, the experimenter annotated the elapsed time (timing). If the response (tracing of the outlines) was wrong, the experimenter continued to take the time until the participant provided the correct response or until 180 s were elapsed. The total time was divided by the number of items (12) to compute the average time (RTs), which was used to measure the individual’s cognitive style. A shorter time indicated a higher predisposition towards field independence, whereas a longer time indicated a higher predisposition towards field dependence.

The Alternative Uses Task (AUT) from the Torrance Test of Creative Thinking TTCT-Form A [62] was employed to measure DT. The AUT aimed to find as many alternative uses as possible for carton boxes within 10 min. The technical manual [62] was used by a single rater who completed a 20 h training on DT to score the alternative uses provided by participants. Only one rater was used because the technical manual clarifies that, if instructions are carefully followed, it is possible to obtain satisfactory reliability scores. Particularly, in this study, the following indices were considered: (1) the number of relevant verbal responses (Fluency—DT-Fluency); the rater was instructed to discard the alternative uses that did not involve a practical use in a specific context (e.g., a carton box can be used to go on the Moon) and retain only those alternative uses that involved a practical value (e.g., a carton box can be used as a carpet). (2) The number of categories encompassing the relevant ideas provided. In order to avoid arbitrary classifications, the rater was instructed to identify the appropriate category for each alternative use by consulting a list of predetermined categories reported in the technical manual; these predetermined categories included a certain number of ideas/elements (e.g., the category ‘furniture’ included the elements ‘bad’, ‘chair’, and so forth). If the idea could not be identified in the categories listed in the technical manual, the rater was instructed to create a new category opportunely that was suitable to encompass the relevant response (Flexibility—DT-Flexibility). (3) The sum of weights of statistically frequent or infrequent responses was provided by the reference sample (Originality—DT-Originality). This latter index was scored as follows: 0 points for responses provided by 5% or more of 500 people; 1 point for responses provided by 2–4.99% of 500 people; 2 points for both responses provided by <2% of 500 people and responses not listed in the technical manual. Furthermore, DT-Fluency, DT-Flexibility, and DT-Originality scores were converted into z-scores and summed to obtain a composite index of DT (DT-Total score) [63]. Previous studies showed that the average scores of the reliability coefficients obtained using six teachers were as follows: fluidity = 0.99; flexibility = 0.95; originality = 0.91. Instead, the reliability coefficients obtained using only one assistant were as follows: fluidity = 0.99; flexibility = 0.98; originality = 0.76 [62].

### 2.3. Statistical Analysis

Analyses were performed using SPSS Statistics version 24 for Windows (IBM Corporation, Armonk, NY, USA). Descriptive statistics were computed to analyze the demographic features of the sample, whereas bivariate correlations were used to check the association among the study variables preliminarily. The moderating role of WMC was tested by the PROCESS macro for SPSS (version 3.5 [64]). Moderation depicts a process in which an independent variable (x) and a third variable (a moderator; w) interact in their influence on the dependent variable (y). Identifying a moderator of an effect allows for establishing the boundary conditions of that effect in terms of large vs. small, present vs. absent, positive vs. negative, and so forth [64]. In this study, the significance of the moderating effect was detected using 5000 resample of bootstrapped estimates with 95% bias-corrected confidence intervals (CIs) [64]. Bootstrapping represents one of several resampling strategies used for estimation and hypothesis testing. In bootstrapping, the sample is considered a pseudo-population that represents the broader population from which the sample was derived [65]. The sampling distribution of any statistic can be generated by calculating the statistics of interest in multiple resamples of the dataset. Additionally, when using bootstrapping, no assumptions about the shape of the sampling distribution are necessary during inferential tests [65]. Bootstrapping is a non-parametric approach that enables an accurate test of the mediation and moderating effects in small- to medium-sized samples, e.g., Refs. [66,67,68,69,70], bypassing the issue of non-normality [71]. The 95% CIs must not cross zero to satisfy the criteria of moderation [64]. All significance was set to *p* < 0.05.

## 3. Results

Data were tested for normality and results showed that all variables are normally distributed (Kolmogorov–Smirnov Test: Z_FDI_ = 0.47, ns; Z_DT-Fluency_ = 0.15, ns; Z_DT-Flexibility_ = 0.08, ns; Z_DT-Total score_ = 0.34, ns) except for WMC and DT-Originality (Kolmogorov–Smirnov Test: Z_WMC_ = 0.00, sig; Z_DT-Originality_ = 0.03, sig). Given the non-normality of some variables, Spearman’s Rho correlations were performed. An analysis showed that FDI was negatively associated with WMC (*r* = −0.30, *p* < 0.01). This suggested that the lower the time in the EFT (i.e., higher disposition toward field independence), the higher the working memory. Regarding demographics, no significant correlations were found with the study variables. Table 1 reports means, standard deviations, and correlations among all variables included in the current research.

**Figure 1:**
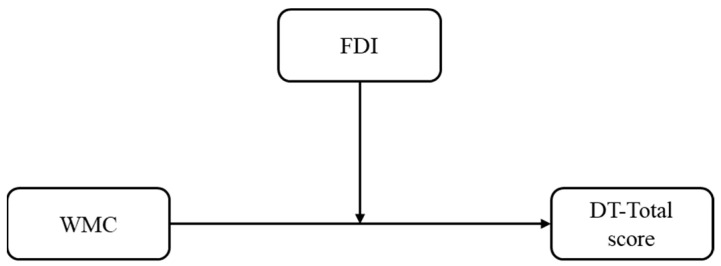
The theoretical moderating model of the study. Note. WMC = working memory capacity; FDI = field-dependent-independent cognitive style; DT = divergent thinking.

**Figure 2:**
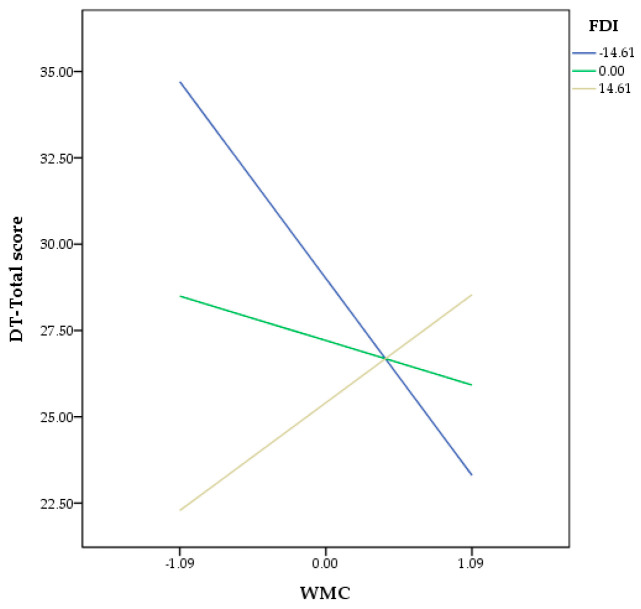
The moderating effect of FDI on the association between the WMC and DT-Total score. Note. WMC = working memory capacity; FDI = field-dependent-independent cognitive style; DT = divergent thinking.

The moderation analysis was performed by entering WMC as the focal predictor, FDI as the moderator, and DT-Total score (DT) as the outcome (Figure 1). No covariates were included in the model (see Table 2). Results showed that the unconditional effects of WMC (B = −0.24, SE = 0.28, t = −0.83, CI 95% = (−0.815, 0.332)) and FDI (B = −0.02, SE = 0.02, t = −1.08, CI 95% = (−0.068, 0.020)) were not significant. However, results showed that FDI moderated the association between WMC and DT-Total score (B = 0.06, SE = 0.02, t = 2.58, CI 95% = (0.0143, 0.108)) only at low levels (B = −14.60, SE = −1.13, t = −3.49, CI 95% = (−1.784, −0.490)). The R^2^ for the entire model was 0.16 (F(3,96) = 5.90, *p* < 0.001). The Johnson–Neyman technique revealed that the threshold for significance of the effect of WMC on DT was located at 32.54 (−4.22 after centring) in FDI. Low values of WMC for the Johnson–Neyman highlighted that the relationship between WMC and DT was significant, whereas above it, WMC was not significant. Overall, these findings suggested that FDI moderated the association between WMC and DT when low levels of FDI (field independence) were involved (Figure 2).

## 4. Discussion

In a sample of 100 adolescents, the present study aimed to investigate the involvement of FDI in the relationship between WMC (span) and DT. We advanced a moderating model, hypothesizing that FI enhanced the effect of WMC on DT. Results confirmed the hypothesis, showing that although WMC did not directly predict DT, the joint effect of WMC and FDI supported adolescents’ DT. These results suggest that DT requires controlled mental processes [39], such as WMC, and the predisposition to acquire, organize, and process information across situations independently from the context. Indeed, FIs show an internal goal orientation and tend to process information analytically, breaking down the problem or the situation into small parts and finding potential relationships between elements. Analysing the problem in detail might facilitate the effect of WMC on DT. By contrast, FDs are externally goal-oriented, tend to process the problem holistically, and do not find potential relationships between elements, likely reducing the ability to recombine remote information into working memory. Additionally, FIs can also manage better cognitive resources (attentional), given that they are more capable of maintaining attention to relevant stimuli than FDs, who, in turn, have more difficulty maintaining their focus and, consequently, are more sensitive to irrelevant stimuli [58]. This means that FI disposition can yield positive effects on WMC and ultimately on DT compared with FD disposition based on individual differences in attentional mechanisms (focused vs. defocused attention). In other words, given that FI involves perceiving and processing relevant vs. irrelevant stimuli, WMC would positively affect DT because the ability to hold and recombine divergently relevant stimuli would be better exploited. However, FIs are better than FDs in using and extracting knowledge from memory, especially when the problem solution is unclear, such as in creative tasks [51]. Thus, it is likely that, concerning FDs, FIs enhance the effect of WMC on DT because they can retrieve and load more information into working memory, increasing the likelihood of getting divergent ideas. FIs are also better than FDs in terms of visuospatial mental imagery. First, FIs are more vivid in their imagination than FDs, creating mental images with more pictorial details [72]. Second, FIs show a higher mental rotation ability than FDs [73]. These results confirm that FIs can also rely on mental images as internal cues. In this vein, it is reasonable to assume that FIs used their knowledge loaded into working memory to create visuospatial mental images that were useful to represent the alternative uses, that is, action simulations of the carton boxes, obtaining a better performance in DT than FDs. Indeed, in the present study, participants were instructed to think of as many uses as possible for carton boxes. Although the task instruction was not focused on mental imagery, participants likely imagined, to some extent, scenarios to figure out alternative uses, mentally representing situations or objects by action simulations in their working memory [74]. Thus, mental representations of the environment define spatial outlines of action possibilities [75]. This implies that FIs can trigger the effect of their WMC on DT by using visuo-spatial mental imagery abilities.

Additionally, these results extend previous studies, highlighting the key role of FDI in the generative/divergent phase of real-world creativity [50] and in the relationship between controlled mental processes and creativity [4,12]. Using the theoretical framework of the Geneplore model [76], triggering WMC, FI would appear to be useful not only in the definition of the pre-inventive ideas, that is, when knowledge is retrieved from memory and is associated and synthesized into a new form, but also in the exploration of pre-inventive ideas, that is, when initial ideas are interpreted by searching for implications, functions, and limitations. Although one would expect that the joint contribution of WMC and FDI differently affect the definition and the exploration of pre-inventive ideas, FI appears to benefit the creative process via WMC based on the ability to shift between a generative (divergent and uncontrolled) phase and an explorative (convergent and controlled) phase. Notably, this view only describes a general cognitive mechanism underlying the creative process. FD might also foster creativity if the creative process is defined by specific social cues (e.g., triggered by brainstorming) [51].

For this study, at least two implications should be highlighted. First, in line with the Investment Theory of Creativity [14], the interplay of individual resources supporting DT could be better understood, including different interacting mental processes. Second, given that the interplay of FI and WMC plays a crucial role in DT, stimulating adolescents to develop the strategies underpinned by FDI (e.g., analysis of the problem, focus on relevant stimuli) could contribute to better exploiting WMC to increase creative potential at school and in everyday life.

Despite these implications, this work is not without limitations. First, a small convenience sample of one hundred adolescents was used. Given this sample size, the study was slightly underpowered. Therefore, the results should be confirmed including larger samples. The key role of other controlled mental processes is lacking, given that only WMC was used as the independent variable. Future studies should also include working memory updating, inhibitory control and shifting in the models to deepen the effects of the interplay between FDI and executive functions on DT. Finally, the creative potential was measured only using a composite score of DT. Future studies should consider a more granulose approach, including convergent thinking tasks and personality measures, for a more reliable measure of creative potential.

## 5. Conclusions

In the present study, the extent to which FDI moderated the WMC-DT link was explored in adolescents. Results showed that FIs better exploit the effect of working memory on DT. In line with the investment theory of creativity [13], this finding shows that the interaction of different individual resources can account for creative potential. This is because FI underpins analytic and associative strategies and the strategy to focus on relevant elements when facing a problem, leading to better exploitation of the knowledge. Thus, FDI increases DT via WMC by associative processes and allocating attention resources.

## Figures and Tables

**Table 1:** Means, standard deviations, and inter-correlations amongst all variables.

	M	SD	1.	2.	3.	4.	5.	6.	7.	8.
1. Age	18.88	0.32	1							
2. Gender			−0.19	1						
3. WMC	4.83	1.09	0.04	−0.09	1					
4. FDI	36.76	14.60	−0.08	0.05	−0.30 **	1				
5. DT-Fluency	9.98	5.00	0.09	−0.07	−0.08	−0.19	1			
6. DT-Flexibility	6.73	2.69	0.09	−0.02	−0.13	−0.12	0.84 **	1		
7. DT-Originality	9.11	6.06	0.07	−0.01	−0.06	−0.16	0.80 **	0.76 **	1	
8. DT-Total score	25.82	12.89	0.08	−0.03	−0.09	−0.16	0.95 **	0.91 **	0.93 **	1

Note. N = 100, gender was dummy coded (0 = F; 1 = M). WMC = working memory capacity; FDI = field-dependent-independent cognitive style; DT = divergent thinking. ** *p* < 0.01 (two-tailed).

**Table 2:** Coefficients for the moderating model of the study.

	B	SE	t	LLCI	ULCI
WMC	−0.24	0.28	−0.83	−0.815	0.332
FDI	−0.02	0.02	−1.08	−0.068	0.020
WMC × FDI	0.06	0.02	2.58	0.014	0.108
*R*^2^ = 0.16					
F(3, 96) = 5.90 ***					

Note. N = 100. SE = standard error, WMC = working memory capacity, FDI = field-dependent-independent cognitive style, LLCI = Lower Limit of the 95% Confidence Interval, ULCI = Upper Limit of the 95% Confidence Interval. *** *p* < 0.001.

## Data Availability

The dataset will be made available upon request.

## References

[1] Cheng V.M.Y. (2019). Developing individual creativity for environmental sustainability: Using an everyday theme in higher education. Think. Skills Creat..

[2] Palmiero M., Piccardi L., Nori R., Palermo L., Salvi C., Guariglia C. (2019). Editorial: Creativity: Education and rehabilitation. Front. Psychol..

[3] Runco M.A., Acar S. (2012). Divergent thinking as an indicator of creative potential. Creat. Res. J..

[4] Giancola M., Palmiero M., Bocchi A., Piccardi L., Nori R., D’Amico S. (2022). Divergent thinking in Italian elementary school children: The key role of probabilistic reasoning style. Cogn. Process..

[5] Guilford J.P. (1967). The Nature of Human Intelligence.

[6] Giancola M., Palmiero M., D’Amico S. (2022). Divergent but not convergent thinking mediates the trait emotional intelligence-real-world creativity link: An empirical study. Creat. Res. J..

[7] Benedek M., Jauk E., Fox K.C.R., Christoff K. (2018). Spontaneous and controlled processes in creative cognition. The Oxford Handbook of Spontaneous Thought Mind-Wandering, Creativity, and Dreaming.

[8] Beaty R.E., Kenett Y.N., Christensen A.P., Rosenberg M.D., Benedek M., Chen Q., Fink A., Qiu J., Kwapil T.R., Kane M.J. (2018). Robust prediction of individual creative ability from brain functional connectivity. Proc. Natl. Acad. Sci. USA.

[9] Ovando-Tellez M.P., Bieth T., Bernard M., Volle E. (2019). The contribution of the lesion approach to the neuroscience of creative cognition. Curr. Opin. Behav. Sci..

[10] Vartanian O., Runco M.A., Pritzker S. (2011). Brain and neuropsychology. Encyclopedia of Creativity.

[11] Witkin H.A., Moore C.A., Goodenough D.R., Cox P.W. (1977). Field-dependent and field-independent cognitive styles and their educational implications. Rev. Educ. Res..

[12] Giancola M., Palmiero M., D’Amico S. (2022). Exploring the interplay between fluid intelligence and creativity: The mediating role of the field-dependent-independent cognitive style. Think. Skills Creat..

[13] Li S., Zhou Y. (2006). The influence of individual cognitive style and material complexity on visuo-spatial working memory. Acta Psychol. Sinica.

[14] Sternberg R.J., Lubart T.I. (1991). An investment theory of creativity and its development. Hum. Dev..

[15] Giancola M., Palmiero M., Piccardi L., D’Amico S. (2021). The contribution of planning to real-world creativity: The moderating role of agreeableness. Think. Skills Creat..

[16] Barbot B., Heuser B., Karwowski M., Kaufman J.C. (2017). Creativity and identity formation in adolescence: A developmental perspective. The Creative Self: Effect of Beliefs, Self-Efficacy, Mindset, and Identity.

[17] Gómez C.M., Barriga-Paulino C.I., Rodríguez-Martínez E.I., Rojas-Benjumea M.Á., Arjona A., Gómez-González J. (2018). The neurophysiology of working memory development: From childhood to adolescence and young adulthood. Rev. Neurosci..

[18] Kerns J.G. (2006). Anterior cingulate and prefrontal cortex activity in an FMRI study of trial-to-trial adjustments on the Simon task. Neuroimage.

[19] Amador-Campos J.A., Kirchner-Nebot T. (1997). Relations of scores on Children’s Embedded Figures Test with age, item difficulty and internal consistency. Percept. Mot. Skills.

[20] Baddeley A.D., Hitch G.J., Bower G.H. (1974). Working memory. The Psychology of Learning and Motivation: Advances in Research and Theory.

[21] Stedron J.M., Sahni S.D., Munakata Y. (2005). Common mechanisms for working memory and attention: The case of perseveration with visible solutions. J. Cogn. Neurosci..

[22] Diamond A. (2013). Executive functions. Ann. Rev. Psychol..

[23] Dygert S.K.C., Jarosz A.F. (2020). Individual differences in creative cognition. J. Exp. Psychol. Gen..

[24] Smeekens B.A., Kane M.J. (2016). Working memory capacity, mind wandering, and creative cognition: An individual-differences investigation into the benefits of controlled versus spontaneous thought. Psychol. Aesthet. Creat. Arts.

[25] Takeuchi H., Taki Y., Hashizume H., Sassa Y., Nagase T., Nouchi R., Kawashima R. (2011). Failing to deactivate: The association between brain activity during a working memory task and creativity. Neuroimage.

[26] Fugate C.M., Zentall S.S., Gentry M. (2013). Creativity and working memory in gifted students with and without characteristics of attention deficit hyperactive disorder: Lifting the mask. Gift. Child Q..

[27] Furley P., Memmert D. (2015). Creativity and working memory capacity in sports: Working memory capacity is not a limiting factor in creative decision making amongst skilled performers. Front. Psychol..

[28] De Dreu C.K.W., Nijstad B.A., Baas M., Wolsink I., Roskes M. (2012). Working memory benefits creative insight, musical improvisation, and original ideation through maintained task-focused attention. Pers. Soc. Psychol. Bull..

[29] Leder J., Hausser J.A., Krumm S., Germar M., Schlemmer A., Kaiser S., Kalis A., Mojzisch A. (2018). The cognitive underpinning of option generation in everyday life decision-making: A latent variable analysis. Cogn. Sci..

[30] Orzechowski J., Gruszka A., Michalik K. (2022). The impact of working memory on divergent thinking flexibility. Think. Reason..

[31] Palmiero M., Nakatani C., Raver D., Olivetti Belardinelli M., van Leeuwen C. (2010). Abilities within and across visual and verbal domains: How specific is their influence on creativity?. Creat. Res. J..

[32] Krumm G., Arán Filippetti V., Gutierrez M. (2018). The contribution of executive functions to creativity in children: What is the role of crystallized and fluid Intelligence?. Think. Skills Creat..

[33] Lee C.S., Therriault D.J. (2013). The cognitive underpinnings of creative thought: A latent variable analysis exploring the roles of intelligence and working memory in three creative thinking processes. Intelligence.

[34] Hao N., Yuan H., Cheng R., Wang Q., Runco M.A. (2015). Interaction effect of response medium and working memory capacity on creative idea generation. Front. Psychol..

[35] Teng J., Shen W., Hao N. (2018). The role of cognitive control in divergent thinking. Adv. Psychol. Sci..

[36] Lu R., Zhang Y., Bao N., Su M., Zhang X., Shi J. (2022). Visuospatial, rather than verbal working memory capacity plays a key role in verbal and figural creativity. Think. Reason..

[37] Wilhelm O., Hildebrandt A., Oberauer K. (2013). What is working memory capacity, and how can we measure it?. Front. Psychol..

[38] Ecker U.K., Lewandowsky S., Oberauer K., Chee A.E. (2010). The components of working memory updating: An experimental decomposition and individual differences. J. Exp. Psychol. Learn. Mem. Cogn..

[39] Benedek M., Jauk E., Sommer M., Arendasy M., Neubauer A.C. (2014). Intelligence, creativity, and cognitive control: The common and differential involvement of executive functions in intelligence and creativity. Intelligence.

[40] Weiss S., Steger D., Kaur Y., Hildebrandt A., Schroeders U., Wilhelm O. (2021). On the trail of creativity: Dimensionality of divergent thinking and its relations with cognitive abilities, personality and insight. Eur. J. Personal..

[41] Zabalina D.L., Friedman N.P., Andrews-Hanna J. (2019). Unity and diversity of executive functions in creativity. Conscious. Cogn..

[42] Lin W.L., Lien Y.W. (2013). The different role of working memory in open-ended versus closed-ended creative problem solving: A dual-process theory account. Creat. Res. J..

[43] Takeuchi H., Taki Y., Nouchi R., Yokoyama R., Kotozaki Y., Nakagawa S., Sekiguchi A., Iizuka K., Hanawa S., Araki T. (2020). Originality of divergent thinking is associated with working memory–related brain activity: Evidence from a large sample study. Neuroimage.

[44] Vartanian O., Jobidon M.E., Bouak F., Nkashima A., Smith I., Lam Q., Cheung B. (2013). Working memory training is associated with lower prefrontal cortex activation in a divergent thinking task. Neuroscience.

[45] Vally Z., Salloum L., AlQedra D., El Shazly S., Albloshi M., Alsheraifi S., Alkaabi A. (2019). Examining the effects of creativity training on creative production, creative self-efficacy, and neuro-executive functioning. Think. Skills Creat..

[46] Riding R., Rayner S. (2013). Cognitive Styles and Learning Strategies: Understanding Style Differences in Learning and Behavior.

[47] Giancola M., Palmiero M., D’Amico S. (2022). Field dependent–independent cognitive style and creativity from the process and product-oriented approaches: A systematic review. Creat. Stud..

[48] Spotts J.V., Mackler B. (1967). Relationships of field-dependent and field-independent cognitive styles to creative test performance. Percept. Mot. Skills.

[49] Niaz M., De Nunez G.S., De Pineda I.R. (2000). Academic performance of high school students as a function of mental capacity, cognitive style, mobility-fixity dimension, and creativity. J. Creat. Behav..

[50] Giancola M., Palmiero M., Piccardi L., D’amico S. (2022). The relationships between cognitive styles and creativity: The role of field dependence-independence on visual creative production. Behav. Sci..

[51] Li C., Mu X., Tan Y., Gu C., Hu B.Y., Fan C. (2023). Do field-dependent individuals tend to have lower creativity than field-independent ones? The role of informational cues in electronic brainstorming. Interact. Learn. Environ..

[52] Lei W., Deng W., Zhu R., Runco M.A., Dai D.Y., Hu W. (2021). Does cognitive style moderate expected evaluation and adolescents’ creative performance: An empirical study. J. Creat. Behav..

[53] Baranovska A., Petlak E., Doktorova D. (2017). Relationship between dimensions of creativity, dependency and independency from the field, need and ability to achieve cognitive closure. Ad Alta J. Interdisci. Res..

[54] Messick S. (1984). The nature of cognitive styles: Problems and promise in educational practice. Educ. Psychol..

[55] Lovano-Kerr J. (1983). Cognitive style revisited: Implications for research in art production and art criticism. Stud. Art Educ..

[56] Witkin H.A., Goodenough D.R. (1981). Cognitive styles: Essence and origins. Field-dependence and field-independence. Psychol. Issues.

[57] Miyake A., Friedman N.P., Rettinger D.A., Shah P., Hegarty M. (2001). How are visuospatial working memory, executive functioning, and spatial abilities related? A latent-variable analysis. J. Exp. Psychol. Gen..

[58] Guisande M.A., Páramo M.F., Tinajero C., Almeida L.S. (2007). Field dependence-independence (FDI) cognitive style: An analysis of attentional functioning. Psicothema.

[59] Al-enezi D.F. (2008). A Study of Learning Mathematics Related to Some Cognitive Factors and to Attitudes. Unpublished Doctoral Dissertation.

[60] Orsini A., Grossi D., Capitani E., Laiacona M., Papagno C., Vallar G. (1987). Verbal and spatial immediate memory span: Normative data from 1355 adults and 1112 children. Ital. J. Neurol. Sci..

[61] Fogliani T., Di Nuovo S., Fogliani A.M., Pizzamiglio L. (1984). Dipendenza dal Campo e Stile Cognitivo: Gli Embedded Figures Tests di H. Witkin, PK Oltman, E. Raskin e SA Karp.

[62] Sprini G., Tomasello S. (1989). Torrance Tests of Creative Thinking (Test di Pensiero Creativo).

[63] Runco M.A., Millar G., Acar S., Cramond B. (2010). Torrance tests of creative thinking as predictors of personal and public achievement: A fifty year follow-up. Creat. Res. J..

[64] Hayes A.F. (2017). Introduction to Mediation, Moderation, and Conditional Process Analysis: A Regression-Based Approach.

[65] Preacher K.J., Hayes A.F. (2008). Asymptotic and resampling strategies for assessing and comparing indirect effects in multiple mediator models. Behav. Res. Methods.

[66] Giancola M., Palmiero M., D’Amico S. (2022). Social sustainability in late adolescence: Trait Emotional Intelligence mediates the impact of the Dark Triad on Altruism and Equity. Front. Psychol..

[67] Giancola M., Palmiero M., D’Amico S. (2023). Dark Triad and COVID-19 vaccine hesitancy: The role of conspiracy beliefs and risk perception. Curr. Psychol..

[68] Giancola M., Pino M.C., Riccio V., Piccardi L., D’Amico S. (2023). Preschoolers’ Perceptual Analogical Reasoning and Map Reading: A Preliminary Study on the Mediating Effect of Spatial Language. Children.

[69] Giancola M., Bocchi A., Palmiero M., De Grossi I., Piccardi L., D’Amico S. (2023). Examining cognitive determinants of planning future routine events: A pilot study in school-age Italian children (Análisis de los determinantes cognitivos de la planificación de eventos de rutina futuros: Un estudio piloto con niños italianos en edad escolar). Stud. Psychol..

[70] Giancola M. (2022). Who complies with prevention guidelines during the fourth wave of COVID-19 in Italy? An empirical study. Pers. Individ. Differ..

[71] Bollen K.A., Stine R. (1990). Direct and indirect effects: Classical and bootstrap estimates of variability. Sociol. Methodol..

[72] Boccia M., Piccardi L., Di Marco M., Pizzamiglio L., Guariglia C. (2016). Does field independence predict visuo-spatial abilities underpinning human navigation? Behavioural evidence. Exp. Brain Res..

[73] Li H., Zhang Y., Wu C., Mei D. (2016). Effects of field dependence-independence and frame of reference on navigation performance using multi-dimensional electronic maps. Pers. Individ. Differ..

[74] Matheson H.E., Kenett Y.N. (2020). The role of the motor system in generating creative thoughts. NeuroImage.

[75] Stevens J.A. (2005). Interference effects demonstrate distinct roles for visual and motor imagery during the mental representation of human action. Cognition.

[76] Finke R.A., Ward T.B., Smith S.M. (1992). Creative Cognition: Theory, Research, and Applications.

